# Dietary profile and phenolics consumption in university students from the Ningxia Hui Autonomous Region of China

**DOI:** 10.1186/s40795-020-00386-z

**Published:** 2020-11-18

**Authors:** Qinghan Gao, Xiao Yuan, Jianjun Yang, Xueyan Fu

**Affiliations:** 1 School of Public Health and Management, Ningxia Medical University, Yinchuan, 750004 Ningxia China; 2 School of Pharmacy, Ningxia Medical University, Yinchuan, 750004 China

**Keywords:** Polyphenols intake, Food sources, University students, Northwest China

## Abstract

**Background:**

Polyphenol intake assessment is a first step for evaluating relationships between polyphenols and health-related outcomes. Ningxia Hui Autonomous Region is one of the minority areas in China, which is primarily consists of arid, dry desert.

**Objectives:**

This study was to make assessment about phenolics intake by university students from Ningxia of China.

**Methods:**

This study employed data from a cross-sectional survey conducted from February to June 2018 in Ningxia Hui Autonomous Region of Northwest China. A total of 413 undergraduate students (143 boys, 270 girls), mean age 20.6 years, participated in the study. Food-frequency consumption and anthropometric measurements were included in the survey. According to phenol-explorer website, the amount of different classes of phenolic compounds were established. Statistics analyses were conducted with IBM SPSS 20.0.

**Results:**

Profile of the student subjects showed low weight (19.1%), overweight (6.8%) and obesity (0.5%). The mean value about phenolics intake was 1378 mg/day. The main polyphenols consumed were flavonoids (58.7% of total polyphenols), followed by phenolic acids (38.1%). Vegetables, fruits and cereals products were the most consumed foods, while infusions and sugar products were lower. Fruit was the main food sources of total polyphenols, especially apple (22.95%), orange juice (19.03%) and apple juice (3.93%).

**Conclusions:**

This is the first study on the polyphenol intake of university students in Ningxia of China. The present results will be benefit for further investigation on the role of polyphenol intake against disease occurrence for this adults group.

## Background

Traditional nutrition intervention has changed reasonably with food to provide health benefits and further prevent deficiencies occurrence. Some food components, such as phenolics as the nonnutrients in traditional nutrition science could provide health benefits. Nowadays polyphenols are best known more than just antioxidants and also playing significant roles in reducing risk of various chronic diseases. Epidemiologists have suggested that phenolics may be against some chronic diseases, such as cardiovascular diseases [1], diabetes [2], cancer [3], and total mortality [4, 5]. In vivo and in vitro evidences also demonstrates the relationship between large amounts of phenolics intake and reduced the risk of dislypidemia, atherosclerosis [6, 7], and inflammatory process refer to cardiovascular diseases [8]. But the previous research on health effects about phenolics mainly comes from the results get in vitro or in animals. Data for humans derived mainly from small trials, always involving in non-physiological polyphenols supplementation [9].

Dietary polyphenols are divided into four major classes, including phenolic acids, flavonoids, stilbenes and lignans, which are comprised of > 500 different compounds with different structures in habitual human diets. Flavonoids are divided into six subfamilies including flavanols, flavanones, flavones, flavonols, isoflavones, and anthocyanidins according to the degree of oxidation of the oxygenated heterocycle which forms part of flavonoids structure [10]. For stilbenes, resveratrol is famous for its anti-carcinogenic effect [11]. Lignans is a group of complex phenolic polymer molecules with phytoestrogenic activity. Certain descriptive data about the intake of polyphenols is already available on the population-level. One previous study invested the work about dietary polyphenol intake of 36,037 adult subjects in Europe (including Denmark, France, Germany, Greece, Italy, Norway, Spain, Sweden, the Netherlands, and the UK), their results showed that factors such as socio-demographic, anthropometric as well as lifestyle were associated with the various intake of polyphenols [12].

Ningxia Hui Autonomous Region is one of the minority areas in China, which is located in northwest of China. Ningxia primarily consists of arid, dry desert. It is one of the poorest provinces in China. The average per-capita income in Ningxia is RMB 3180, which was 23% below the mean national income [13]. Up to now, there is no descriptive total polyphenol intake study available in Ningxia, especially for university students there. College life is a critical period that can affect adult chronic diseases, so dietary intake during college may indicate adult lifestyle and health. Polyphenol intake assessment is a first step for evaluating relationships between polyphenols and health-related outcomes. Therefore, the evaluation of dietary patterns and identification of the intake of polyphenols among university students in Ningxia is important and might be helpful for interventions in promoting healthy eating behavior and preventing dietary-related diseases.

The present work was to estimate dietary polyphenols intake, and the main food contributions with food-frequency consumption and the Phenol-Explorer 3.0 for university students from China northwest [14]. This is helpful for promoting health education and public health for this group population. Knowledge of phenolics intakes among university students also might be useful for planning targeted prevention strategies at an early acceptable age.

## Methods

### Participants

This study employed data from a cross-sectional survey conducted from February to June 2018 in Ningxia Hui Autonomous Region of Northwest China. A total of 413 undergraduate students (143 boys, 270 girls), mean age 20.6 years, participated in the study. The whole protocols study with students was fully approved by the scientific committee of Ningxia Medical University. All the students participated in the protocols were informed and consent for future use of questionnaire data. Subjects were informed of the aim of the research, and if they would like to participate, and collecting data with personal appointment.

### Nutritional survey

A nutritional survey was conducted by trained staff with food frequency questionnaires (FFQ) and anthropometric measurements. A total of 49 food items were included in the FFQ, covering nine food groups (fruits, vegetables, infusions, cereals, dairy, bakery, sugars, meats and oils). The FFQ was examined by our skillful registered dietitians. The present FFQ used for polyphenol intake was established according to our previous validated FFQ in university students to assess the consumption frequency of foods and beverages, which were rich in polyphenols [15]. Phenol-Explorer 3.0 was applied for calculating polyphenol contents [14]. The energy calculated based on the China Food Composition Table [16]. During survey process, photo food models were used in order to standardize the types and amounts of the main food groups, such as fruits, vegetables, cereals, sugar, infusions, spoons, cups, as well as plates [17]. Physical activity has been defined the World Health Organization that any body movement produced in leisure time by skeletal muscles requiring energy consumption. In the present study, physical activity levels of the participants were classified into sedentary and moderated activity. The sedentary was the person reported with no leisure activity or done some activity not meeting the regular level. Moderated activity indicated moderate physical activities included yoga, walking at a brisk pace, cycling at a normal speed and lifting light objects [18].

### Anthropometric measurements

According to the World Health Organization guidelines, anthropometric measurements were performed [19]. Weight and height were measured with electronic scales (Hochoice, EF07, China).

### Estimation of polyphenol intake

According to Food Composition Tables in phenol-explorer website, the amounts of phenolic compounds belonging to different classes were established (http://www.phenol-explorer.eu) [14]. For more than 400 foods, 35,000 values of 500 different polyphenols presented in Phenol-explorer website. More than 60,000 values phenolics content from more than 13,000 publications were critically evaluated, and then were included in the Phenol-explorer.eu database [12, 20]. Total polyphenol contents were calculated as the result of summing up the contents of individual compounds and expressed in mg/100 g food weight. Foods such as noodles and orange fruit, their missing polyphenol contents can be extrapolated from wheat flour and orange juice, respectively.

### Statistical analysis

Categorical variables including anthropometric results and physical activity level were showed as percentages. Continuous variables such as age, energy intake and basal metabolism rate (BMR) were presented as means ± standard deviations (SD). Each food group contribution to the total phenolics intake and polyphenol subfamily classes was also showed as a percentage. The general linear model was used to analyze the intake of total polyphenols and polyphenol classes. *P* < 0.05 was set up as the significant level. Statistics analyses were conducted with IBM SPSS 20.0.

## Results

**Fig. 1:**
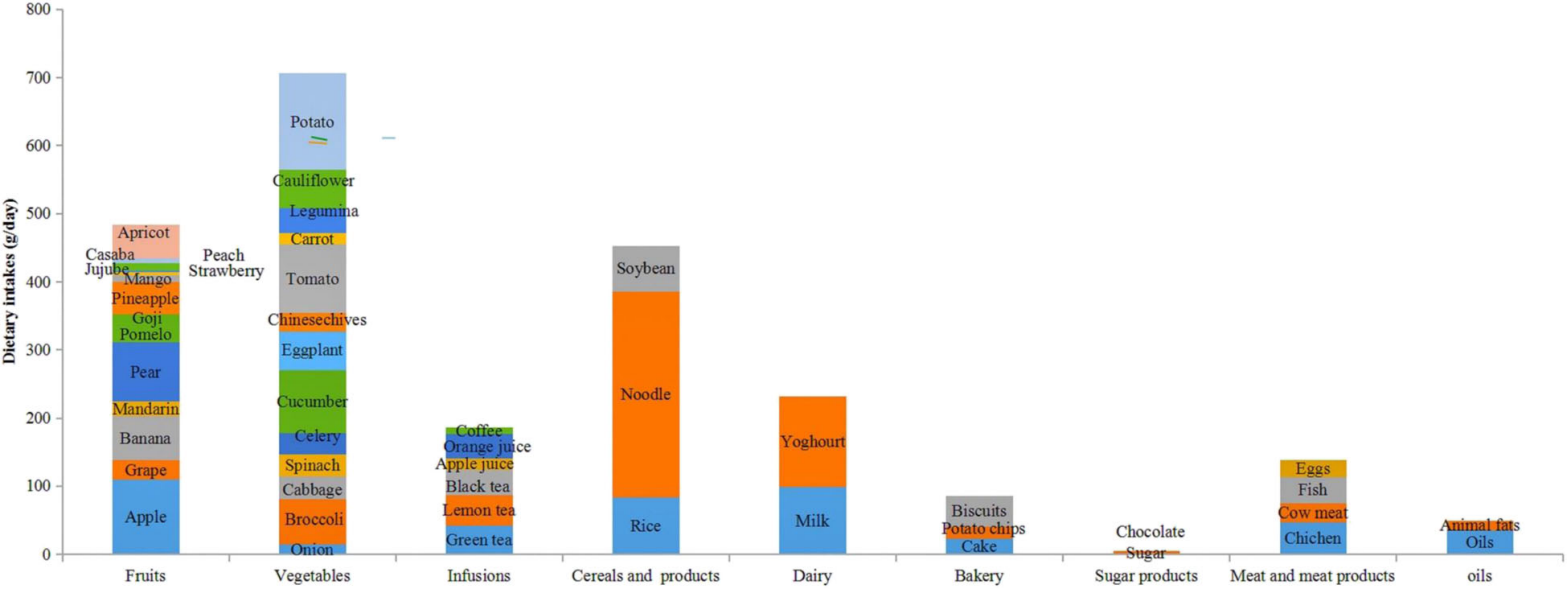
Mean daily intake of foods by university students from the Minority Area of China

Profile of 413 university students are shown in Table 1. From anthropometric results, it can be found that low weight and obesity were coexisted. Referring to eating habits, this northwest region population has made remarkable progress in consuming industrialized foods instead of traditional preparation. Food consumption groups were shown in Fig. 1. Vegetables, fruits and cereals products were the three most consumed foods, while other important food groups such as infusions and sugar products were consumed less. From Table 2, it can be found that the main food sources of total polyphenols was fruits, especially apple (22.95%), non-alcoholic beverages such as orange juice (19.03%) and apple juice (3.93%).

**Table 1:** Profile of the 413 university student subjects

	Total	Boys	Girls	*P*
Age (y)	21.0 ± 1.7	21.0 ± 1.6	21.3 ± 1.7	0.084^a^
Energy intake (kcal/d)	1871 ± 626	1831 ± 576	1893 ± 647	0.163^b^
BMR (kcal/d)	1473 ± 176	1683 ± 117	1362 ± 166	0.000^b^
BMI-for-Age (%)				0.000^b^
Low weight	19.1	8.4	24.8	
Overweight	6.8	14	3.0	
Obesity	0.5	0.0	0.7	
Physical Activity (%)				0.426^b^
Sedentary	10.2	9.1	10.7	
Moderated activity	89.8	90.9	89.3	

*BMR* Basal metabolic rate (kcal/d)

Values expresses as mean ± SD or %

^a^T studen’s test

^b^Kruskal-Wallis test

**Table 2:** Contribution of the main sources of polyphenol content in the diets of university students

Food	Mean intake (g/d)	Polyphenols content (mg/100 g)	Polyphenols contributed by mean intake (mg)	Relative contribution (%)
Apple	109.15	296.3	323.41	22.95
Grape	28.66	201.0	57.61	4.09
Banana	65.03	90.4	58.79	4.17
Pear	87.43	71.0	62.08	4.41
Pineapple	47.16	94.3	44.47	3.16
Broccoli	66.39	101.6	67.45	4.79
Spinach	32.76	91.0	29.81	2.11
Cucumber	91.54	19.5	17.85	1.27
Eggplant	56.61	87.0	49.25	3.49
Tomato	100.87	68.0	68.59	4.87
Potato	142.31	38.0	54.08	3.84
Green tea	42.32	65.8	27.85	1.98
Black tea	37.33	80.5	30.05	2.13
Apple juice	16.37	339.0	55.49	3.93
Orange juice	35.53	755.0	268.25	19.03

**Fig. 2:**
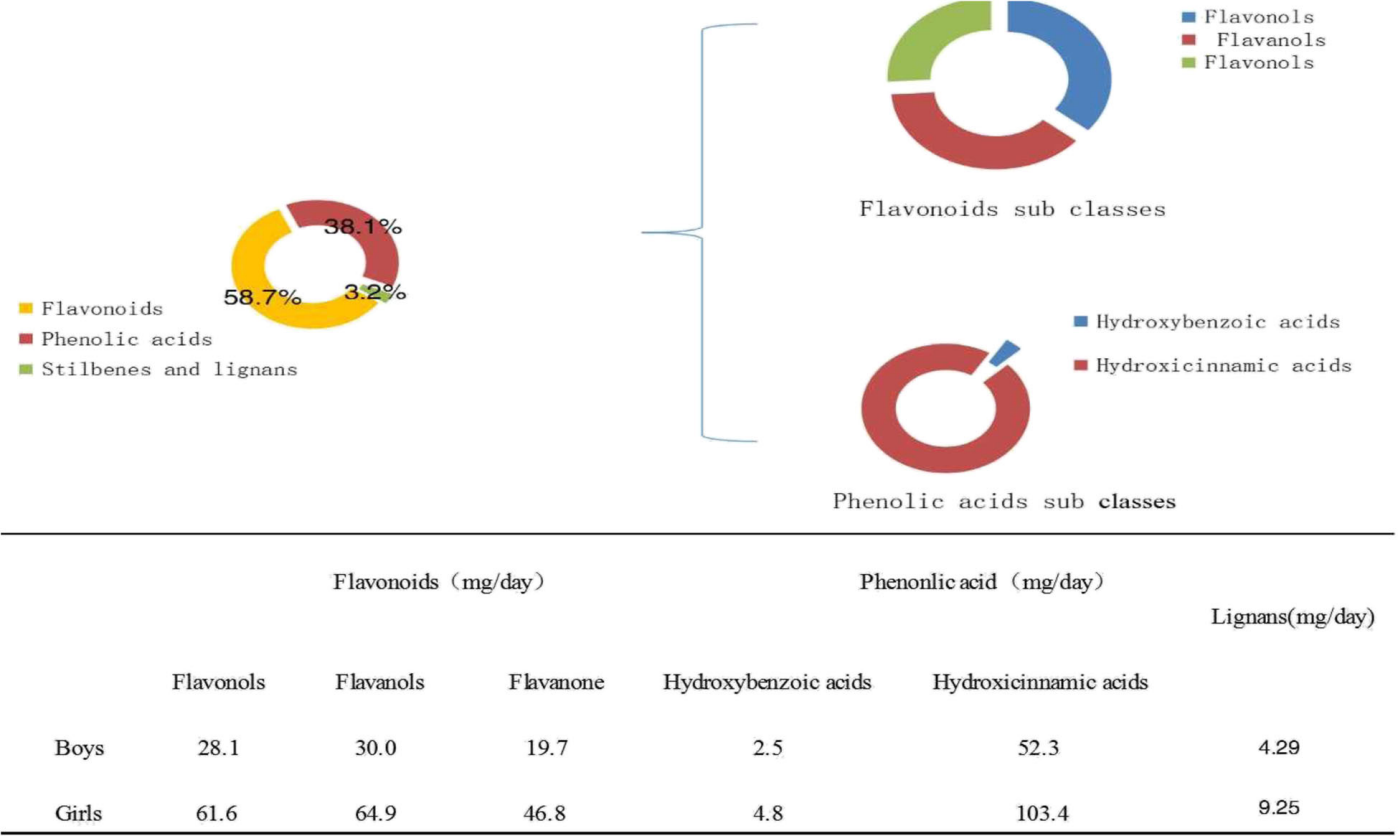
Phenolic compounds intake, classes and sub classes distribution

The mean value of phenolics intake in these students was 1378 mg/day. The distribution about the classes and subclasses of polyphenols was shown in Fig. 2. Flavonoids (58.7%) contributed most for the total intake of phenolics. Phenolic acids contributed 38.1% of total intake of phenolics. Stilbenes and lignans accounted for < 3.2% of total polyphenol intake, and the mainly food sources were cereal products, whereas selected fruits and vegetables account of a smaller proportion for university students in China northwest. The present results were in line with Tetens et al. [21].

With regard to polyphenol subclasses, the three most important contributors to flavonoids subclasses intake were flavanols (38%), flavonols (36%) and flavanone. Hydroxycinnamic (96%) and hydroxybenzoic acids were the two most contributors to phenolic acids. The values of polyphenol intake were higher in girls when compared with boys.

Table 2 list of the main sources of phenolics and the relative contribution of different foods item. In university students from the minority area of China, fruit and vegetables were the two important polyphenol contributors. Among them, apple (22.95%) and orange juice (19.03%) were the major dietary sources of polyphenols.

## Discussion

Lifestyle and eating pattern have changed remarkably, which could be due to the progress made in technology and communication systems over the last decades [22].

Polyphenol intake was comparable with the mean intake in some Mediterranean countries, and similar the European Prospective Investigation into Cancer and Nutrition-Greece (1626 mg/day for women in Denmark) [12].

Flavonoids were mainly contributed by fruits and vegetables consumed for university students in Ningxia. Zhang et al. Estimated that the mean flavonol and flavone intakes were 14.30 mg/day and 4.82 mg/day, respectively in Chinese Adults [23]. One previous study suggested that intakes of total flavonoids, flavan-3-ols, flavanones and anthocyanidins were negatively associated with the risk of high CVD risk in USA adults [24, 25]. Feliciano group suggested that people who have a high consumption of flavonoids (≥ 788 mg/day) could reduce the risk of all-cause mortality [26].

Phenolic acids were in consistence with former study, hydroxycinnamic acids were the most consumed subclass of phenolic acids. In this studied population, the intake of phenolic acids is lower than other research groups reported [27]. Phenolic acids were mainly contributed by infusions such as coffee and tea for university students of Ningxia.

Fruit and vegetables were the two important polyphenol contributors. One research in Chinese adults also reported that apple was the main food sources of flavonols and flavones [23]. The present results is in line with the Australian population where apple was the important flavonoid source for young people [28], and the contribution of fruit to flavonoid intake was higher than that for vegetables [29].

Although some studies found that coffee was the major food source of polyphenols [24, 30, 31], this was not the case in this studied population, as students drink less coffee in this place. In addition, one previous study also showed similar result that coffee contributed less about polyphenols, but fruit juice, sugar-sweetened beverage and water contributed much [32]. This could give the reason why fruit juices were the second largest contributions of polyphenols in this study.

Based on a recent study, the researchers identified median total polyphenol intake was found to be the lowest in Yucatan (536 mg/day) and the highest in Baja California (750 mg/day). The two main phenolics consumed were phenolic acids (56.3–68.5% of total polyphenols) and flavonoids (28.8–40.9% of total polyphenols) [33]. A previous study carried out in Italy reported that people with type 2 diabetes consumed 683.3 ± 5.8 mg/day of phenolics. The main dietary polyphenols source were non-alcoholic beverages, which account of 35.5% of total phenolics intake, followed by fruits (23.0%), alcoholic beverages (14.0%), vegetables (12.4%), tubers and cereal products (4.6%), legumes (3.7%) as well as oils (2.1%). Chocolates, cakes and nuts were three negligible sources of polyphenols in this cohort. Flavonoids (47.5%) and phenolic acids (47.4%) contribute much to the total phenolics intake [34]. Another study from Brazil revealed that the mean total phenolics intake was 1198.6 mg/day. Phenolic acids (729.5 mg/day) and flavonoids (444.7 mg/day) also were the two main polyphenol classes. Coffee (45.8%), beans (32.8%) and polenta (1.3%) were the three larger dietary contributors for total phenolics consumed there [35]. For the group of elderly Japanese, their average total polyphenol intake was around 1492 mg/day, of which beverages (79.1%) contributed as the greatest part [36].

Compared to previous studies in which men had higher total polyphenol intake than women [15, 22], especially for flavonoids [37], girls had a higher intake than boys in this study.

At present, although modernization happened in some regions of the world, healthy eating styles are maintained such as the mediterranean daily diet, which contains foods rich in phenolics. The updated dietary guidelines showed herbal infusions, fruits and vegetables are at the base. Therefore, it proposes educational strategies for further implementation of the updated recommendations. From this aspect, nutritional education should take actions and incorporate with food and this will be benefit for the young population.

Despite of different studies on polyphenols intakes were done in some countries, no previous work of this kind was done in China. Up to now, there is no descriptive total polyphenol intake research available in Ningxia, which is located in China northwest, especially for university students there. College life is a critical period that can affect adult chronic diseases, so dietary intake during college may indicate adult lifestyle and health. It is a first step for evaluating relationships between phenolics and health-related outcomes through proper evaluation of phenolics intake.

Two strengths of this study were the special participants and the use of Phenol-Explorer, which is an extensive food composition database and include a complete list of 500 polyphenols. But there was also some limitations in our study. It was likely underestimation of true phenolics intake due to miss some regional foods data. All subjects involved in this study were university students and therefore very low education people or adults were recruited, so the present results cannot be totally generalisable.

## Conclusions

Intake of total polyphenol was 1378 mg/day. Main food sources of polyphenols were fruits, especially apple (22.95%), orange juice (19.03%) and apple juice (3.93%). These results show significant importance of promoting a healthier diet intake in young adults with more fruits, vegetables and nuts to increase the currently relatively lower polyphenol intakes. Research on young populations intakes of polyphenols will be useful for recommendations establishment for this group. The present results will be benefit for further investigation on the role of polyphenol intake against disease occurrence for young population.

## Data Availability

The datasets used and/or analysed during the current study are available from. the corresponding author on reasonable request.
